# Study protocol: assessing SleeP IN infants with early-onset atopic Dermatitis by Longitudinal Evaluation (The SPINDLE study)

**DOI:** 10.1186/s12887-022-03382-3

**Published:** 2022-06-18

**Authors:** Cathal O’Connor, Alan D. Irvine, Deirdre Murray, Michelle Murphy, Jonathan O’B Hourihane, Geraldine Boylan

**Affiliations:** 1 Department of Paediatrics and Child Health, Cork University Hospital, Cork, Ireland; 2 Department of Dermatology, South Infirmary Victoria University Hospital, Cork, Ireland; 3 INFANT research centre, University College Cork, Cork, Ireland; 4 Department of Dermatology, Children’s Health Ireland at Crumlin, Dublin, Ireland; 5 Department of Clinical Medicine, Trinity College Dublin, Dublin, Ireland; 6 Department of Medicine, University College Cork, Cork, Ireland

**Keywords:** Atopic dermatitis, Sleep, Sleep disruption, Electroencepalography, Actigraphy, Sleep diaries, Parental sleep disruption, Early intervention

## Abstract

**Background:**

Atopic dermatitis (AD) is the most common chronic inflammatory skin condition in childhood. Most (50-60%) children with AD report sleep disturbance, which is secondary to itch, dry skin, inflammation, and abnormal circadian rhythm. Sleep is essential for brain development, learning, and growth. Sleep disruption in early life is associated with cognitive and psychological dysfunction in later life. The aim of this study is to describe in detail the sleep architecture of infants with early-onset atopic dermatitis (AD), compared to controls, by using EEG polysomnography, sleep actigraphy, and parental reporting.

**Methods:**

This observational study will recruit six- to eight-month old infants with moderate to severe AD and age-matched control infants who do not have AD. At six-eight months diurnal sleep electroencephalography and polysomnography will be performed in our research center. Nocturnal sleep actigraphy will be performed at home for five consecutive nights at six-eight months and 12 months. Between six and 12 months, monthly questionnaires will capture data on quantitative sleep and parental sleep. Skin barrier and immune profiles will be captured at six-eight and 12 months. AD will be assessed using standardized severity assessment tools and treated according to protocol. A neurodevelopmental assessment will be performed at 18 months to assess cognition and behaviour. An estimated sample size of 50 participants in each group is required to power the primary outcome of disturbed macrostructure of sleep and secondary outcomes of disturbed microstructure of sleep, and disturbed parental sleep, assuming an attrition rate of 60%. Potential confounding factors which will be controlled for in the data analysis will include parental educational level, parental depression, feeding practice, and number of siblings.

**Discussion:**

This study will provide a rich analysis of sleep in infants with AD in the first year of life using detailed electroencephalography, novel actigraphy techniques, and longitudinal parent-reported data. It may provide guidance on the optimal treatment of AD to prevent or reduce sleep disruption.

**Trial registration:**

clinicaltrials.govNCT05031754, retrospectively registered on September 2nd, 2021.

## Background

Atopic dermatitis (AD) is the most common chronic inflammatory skin condition in childhood. Our group has published the Irish national data reference set that shows that 18.6% of Irish children develop AD by 6 months of age, and 2.4% of children develop severe AD by 6 months [1]. Uniparental history of AD is associated with an odds ratio of 1.7 for AD in offspring, and diparental history of AD is associated with an odds ratio of 2.7 [2]. AD has a complex aetiology, characterized by a dysfunctional skin barrier, dysfunctional immune responses, and dysbiosis (alteration of microbiome from normal composition and function) [3]. It is associated with multiple co-morbid conditions such as food allergy, allergic rhinitis, asthma, anxiety/depression, and attention deficit hyperactivity disorder [4]. It is associated with huge economic costs [5] and poor quality of life indices [6]. Children with severe AD have a worse quality of life than children with asthma, epilepsy, or diabetes [7]. Itch and sleep disturbance are the most burdensome symptoms of AD [6].

Healthy sleep is vital for good health, and can generally be defined as attaining adequate sleep duration, good sleep quality, maintaining appropriate sleep timing, and having no sleep disorders [8]. Sleep in young children is essential for development of the hippocampus, brainstem, and midbrain [9] and for learning [10]. In infancy, extensive sleep is required for brain development and for optimizing physical growth. Sleep is also a fundamental prerequisite for brain plasticity, the genetically determined ability of the brain to change its structure and function in response to stimuli in the environment. In young animals, is has been shown that sleep deprivation can lead to increased neuronal apoptosis, smaller brain volume, and reduced brain plasticity, all of which have persistent deleterious repercussions on behavior and ability to learn [11–13]. Childhood sleep disorders have been associated with less white matter microstructural integrity in adolescence [14]. It has been shown that sleep quality at 10 months accounts for a significant percentage of variance in cognitive achievement [15].

Most (50-60%) children with AD report sleep disturbance [16, 17]. Sleep disturbance is a significant burden in AD and a key indicator of severity [18, 19]. AD has been demonstrated to have a negative impact on both sleep duration and quality of sleep [20–22]. Sleeplessness is also a major factor leading to impaired quality of life [7, 23]. The mechanism of sleep disturbance in patients with AD is complex and pruritus is not the sole cause [24]. Alterations in circadian rhythm, immune dysregulation and increased transepidermal water loss (TEWL) are also thought to play a role [25, 26]. Children with AD have disrupted sleep even when the dermatitis has been treated [27]. AD not only impacts on the sleep of patients but has also been shown to have a negative impact in caregivers [28]. Caregivers of patients with AD often report poorer quality of sleep, increased symptoms of insomnia and chronic sleep deprivation [29]. Co-sleeping is reported by 30% of families with a child with AD due to poor sleep [17].

Data on sleep disturbance in the first year of life in AD are extremely limited. No high-quality research using neurophysiological assessment of sleep disturbance has been performed in infants with AD under 12 months [30, 31]. In addition, very little research has been performed in sleep in infants or children under 3 years with AD. Sleep disruption in AD has generally been measured with actigraphy (measurement of movement) or caregiver-reported measures. However, polysomnography which uses electroencephalography (EEG) and other physiological signals is considered the gold standard of assessment of sleep quality [32]. Video EEG in addition to polysomnography has never been used to provide a detailed assessment of sleep quality in infants with AD.

**Fig. 1:**
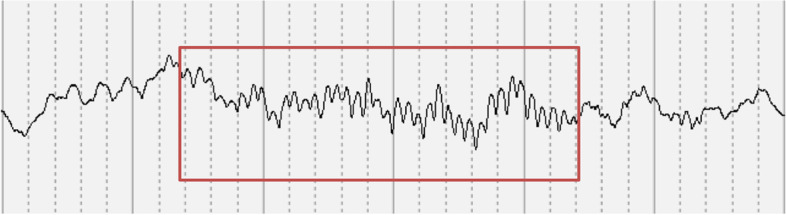
Data from the ENRICH study showing sleep spindles in a 4-month-old infant. Sleep spindles highlighted in red

In the ENRICH study (www.enrichstudy.com) we have recently shown the importance of EEG analysis of sleep, particularly features of sleep microstructure e.g. sleep spindles. We found that healthy infants at 4 months who had participated in a regular daily massage routine had higher power in their sleep spindles than control infants (paper submitted for publication) (Fig. 1). Sleep spindles are associated with cortical plasticity and memory consolidation and are a marker of brain maturation. In older children, increased power has been shown to be positively correlated with processing speed and cognitive ability [33, 34].

In children with AD, disturbed sleep has been associated with behavioural deficits such as Attention Deficit Hyperactivity Disorder [35], but its impact on neurocognitive performance is unknown. Given that AD disturbs sleep, and that disturbed sleep has been shown to reduce neurocognitive performance, AD in infancy may be associated with impaired neurocognitive development. The more severe the AD, the greater the sleep deficit may be, and conceivably, therefore the worse the impact on behaviour and neurodevelopment. Early intervention and treatment of AD may represent an opportunity to prevent sleep disturbance and thereby prevent subsequent developmental problems.

AD is associated with skin barrier defects, resulting in increased TEWL and increased entry of antigens and pathogens. Filaggrin (*FLG*) mutations are the commonest genetic predisposition to the development of AD [36]. Filaggrin is a structural protein critical to corneocyte formation in the stratum corneum. In healthy skin, filaggrin is degraded by proteases into natural moisturizing factors (NMF). Skin barrier function can be assessed by TEWL or by NMF measured by Raman spectroscopy, with reference data previously published by INFANT [37, 38]. Higher TEWL and lower NMF levels are associated with more severe AD [39, 40].

Tape stripping the stratum corneum offers a non-invasive means of assessing inflammatory cytokines in skin, with higher levels correlating to higher disease severity [41]. Th2-skewed cytokines such as IL-13 have been shown to be raised both in the stratum corneum and in serum [42]. The relationship between inflammatory cytokines in AD and impact on sleep is unclear [24].

### Study objectives

The research question of this study is: do infants with early-onset AD have disrupted quantity and quality of sleep in the first year of life, compared to healthy controls, and does the degree of sleep disruption correlate with the severity of AD? Our hypothesis is that infants with early-onset AD have impaired quantity and quality of sleep in the first year of life, which correlates with the severity of AD.

The aim of the project is to describe, for the first time, detailed sleep architecture of infants with early-onset atopic dermatitis (AD), compared to controls, by using EEG polysomnography, sleep actigraphy, and parental reporting.

The objectives are to:

1. Recruit a cohort of infants with early-onset AD and a cohort of matched healthy controls
2. Collect and analyze parent-reported, actigraphy, and EEG polysomnography sleep data from infants with early-onset AD, and compare to healthy controls
3. Perform serial clinical assessment, skin barrier assessment, and inflammatory cytokine assessment (by tape stripping), and correlate with sleep features in infants with early-onset AD
4. Examine sleep and quality of life indices of caregivers of infants with early-onset AD compared to controls

## Methods/design

**Fig. 2:**
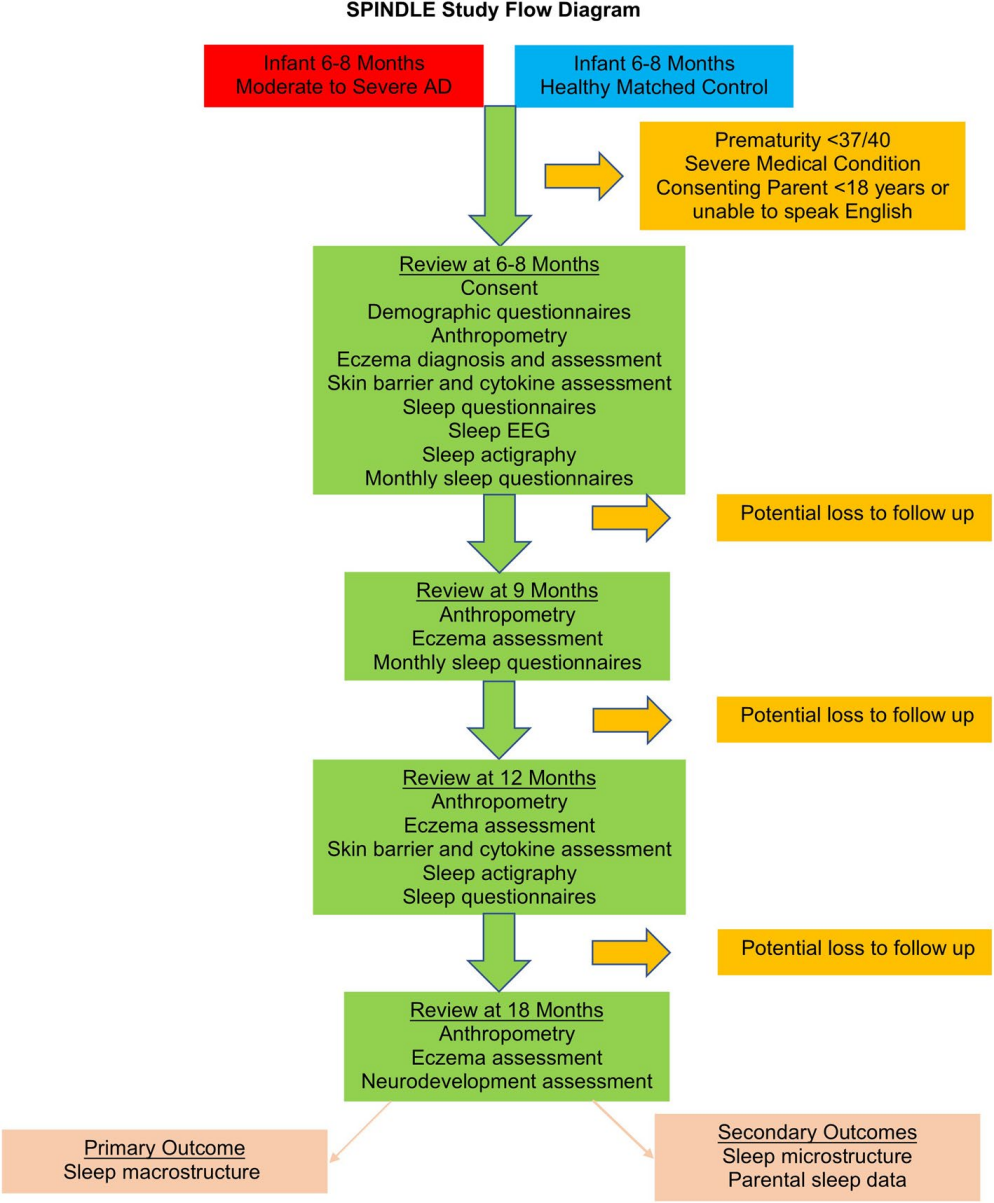
SPINDLE study flow diagram. EEG = electroencephalogram

The study will be observational with no intervention and four study visits in total (Fig. 2). Subjects will be recruited as referrals with AD from primary care and secondary care. The study will be advertised through Cork University Maternity Hospital (CUMH), a tertiary maternity center; the local pediatric unit in Cork University Hospital (CUH); and local general practice surgeries. It will also be advertised on social media, on the INFANT website, and to University College Cork (UCC) staff and students. A contact phone number and email address will be advertised to facilitate rapid referral. Ethical approval has been obtained from the Clinical Research and Ethics Committee of the Cork Teaching Hospitals.

Infants aged six-eight months of age diagnosed with moderate to severe AD are eligible for the study. The parent or guardian must be at least 18 years of age and the consenting adult must be able to speak English. Exclusion criteria include premature birth (defined as delivery before 37 weeks’ gestation), severe medical condition (such as epidermolysis bullosa or ichthyosis) at time of enrolment that would complicate assessment for AD or preclude the use of investigational tools, or other major health problems (especially genetic or metabolic) that would have implications for neurodevelopment. Any medical condition requiring the using of sedating medications (e.g. antihistamines) will be excluded.

Healthy control infants, matched in age, will be recruited for the study by advertising through the maternity hospital, the INFANT website, social media platforms and networking. We have previously recruited a large healthy control cohort of infants to a sleep study (the ENRICH study) and found that parents and guardians are very happy to participate in studies that examine sleeping habits in infants. Infants with a parental history of atopic dermatitis, asthma, or allergic rhinitis will be excluded from the study as controls.

At the first visit at six-eight months of age, infants will be assessed in the INFANT Research Centre in CUH for suitability for entry to the study. The study will be discussed with the parent(s)/guardian(s). If written consent is provided, a baseline assessment will be made. Anthropometric assessment will involve measurement of weight, length, and head circumference. All parents will be asked to complete detailed questionnaires about demographics, environmental factors (home location, ambient bedroom noise, number of siblings), parental educational attainment, feeding practices, and family history of atopic disease. The Maternal Post Natal Attachment Scale and The Edinburgh Post Natal Depression Scale will also be completed at this visit. These questionnaires will collect data about potential confounding factors. Parents will be asked about specific sleep problems with a thorough history of the infant’s 24-hour routine, focusing on naps and sleep routine, sleep environment (crib or parent’s room), sleep position (prone or supine), and need for sleep aids (eg soother, rocking, patting). Parents will be asked to complete the Pittsburgh Sleep Quality Index, a self-report questionnaire that assesses sleep quality over a one-month time interval [43].

For cases, AD will be diagnosed using the Hanifin and Rajka criteria [44]. Severity will be assessed by the Investigator Global Assessment (IGA), Eczema Area and Severity (EASI), and Scoring Atopic Dermatitis (SCORAD) scores [45, 46]. Moderate AD will be defined as IGA 3, EASI 7-12, or SCORAD 15-40. Severe AD will be defined as IGA 4, EASI > 12, or SCORAD > 40. Symptoms and quality of life will be assessed using the Patient-Orientated Eczema measure, Pruritus Numerical Rating Scale with a visual analogue scale, and Infants’ Dermatitis Quality of Life Index [47–49]. The influence of AD on parental quality of life will be assessed using the Dermatitis Family Impact Questionnaire [50]. Previous treatments for AD will be documented.

**Fig. 3:**
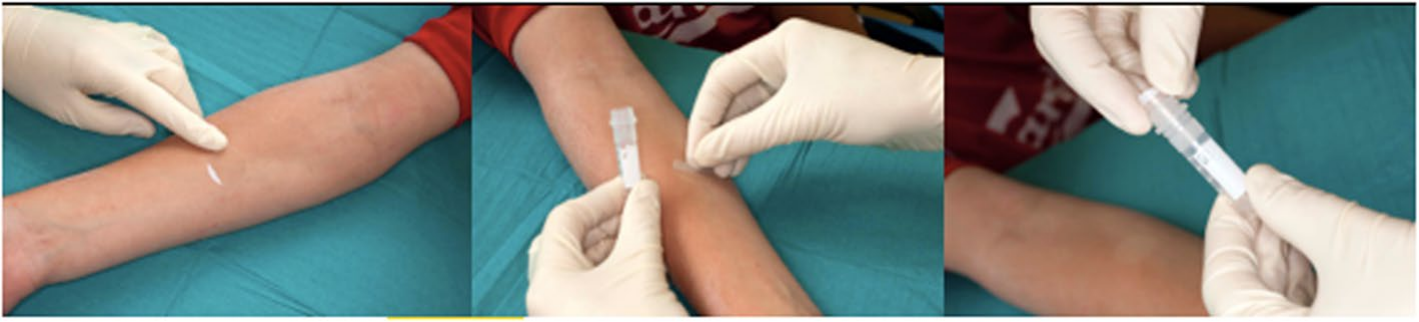
Sampling of inflammatory cytokines from the stratum corneum by tape stripping

All subjects will have transepidermal water loss (TEWL) assessed [37]. TEWL is defined as the loss of water through the epidermis to the surrounding atmosphere via diffusion and evaporation. It can be measured with a device containing two moisture sensors and two thermometers that calculate the rate of evaporation. All subjects will also have measurements of natural moisturizing factor (NMF) via Raman spectroscopy [38]. A skin sample using tape stripping will be taken for inflammatory cytokine analysis. This can be measured by lifting off the outer layer of skin with small (2 cm diameter) adhesive discs like Sellotape (Fig. 3). Tape stripping is performed on non-lesional skin, 2 cm away from viable eczematous areas, and immediately stored at − 80 °C There is a standard operating procedure in use already for sample collection, transport and storage. Cytokine assays will be performed as a batch once patient visits have been completed. A buccal cell swab will be collected from children with AD at the six-eight, nine, or twelve month visit to assess for FLG mutations.

All participants will receive general skin-care advice to avoid soap and bubble bath, use mild cleansers and shampoos that have been specifically designed for babies, and avoid using baby wipes based on NICE guidance on infant skin care [51]. Treatment will be prescribed for babies with AD, as per standard protocols. Parental usage of products will be recorded with eczema treatment diaries. Contact will be maintained with parents by telephone or email. A contact mobile phone number and email address will be supplied to each subject. If skin problems develop during the study, parents will contact the study researcher.

**Fig. 4:**
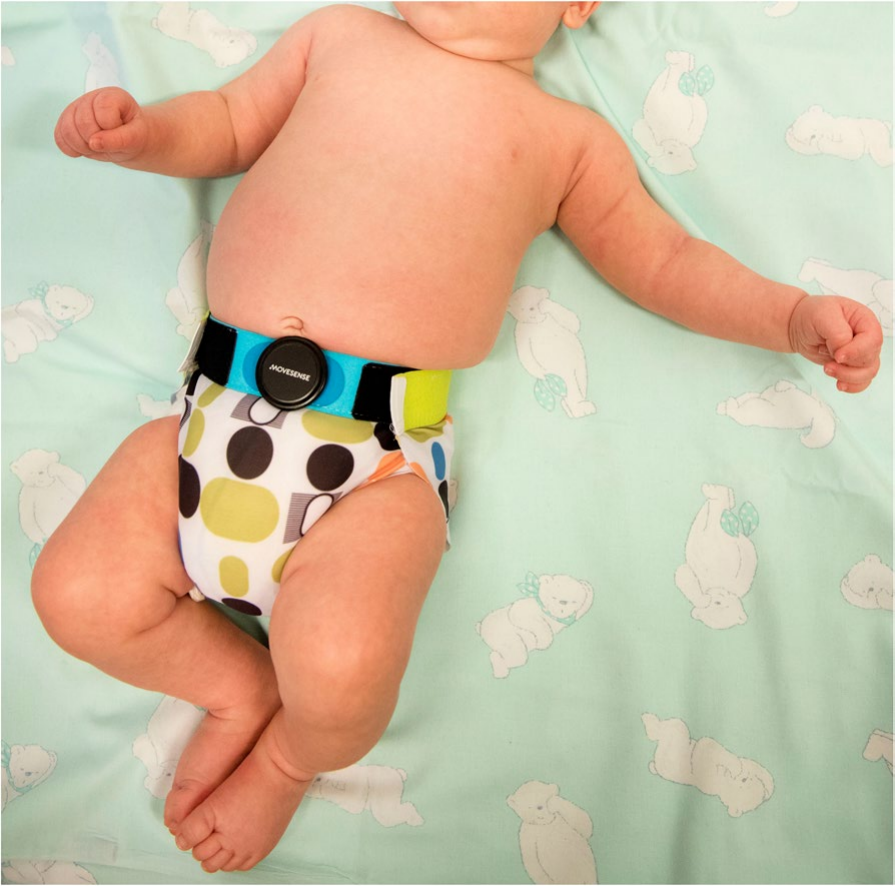
Sleep pants with belt to attach sensor device for overnight actigraphy

In the same visit, a sleep video/EEG will also be performed at the INFANT sleep laboratory (Fig. 4). The timing of this should be coordinated so the recording commences following a feed around the time of the baby’s longest daytime nap. The recording will last for the duration of the baby’s sleep. A multichannel video EEG will be performed using the 10-20 system of electrode placement. Electrooculography, electrocardiography, respiration monitoring, and electromyography will be recorded simultaneously. The following parameters will be measured: duration of rapid eye movement (REM) and non-REM sleep stages, REM onset latency, sleep spindle number, density, frequency, duration, and spectral power. A quantitative analysis of the sleep EEG will be performed using multiple features of amplitude, spectral shape, continuity, and inter-hemispheric connectivity using the NEURAL software [52].

Each parent will be provided with a pair of sleep actigraphy pants with an embedded sensor belt to monitor overnight sleep movements every night for five nights following the visit (Fig. 4).

Parents/guardians will be asked to complete monthly sleep diaries and questionnaires at seven and 8 months to collect data on the infant’s 24-hour routine, focusing on naps and sleep routine, sleep environment (crib or parent’s room), sleep position (prone or supine), and need for sleep aids (e.g. soother, rocking, patting).

At 9 months, weight, length, and head circumference will be measured again. Parents will again be asked about specific sleep problems by thorough history of the infant’s 24-hour routine, focusing on naps and sleep routine, sleep environment (crib or parent’s room), sleep position (prone or supine), and need for sleep aids (e.g. soother, rocking, patting). Parents will be asked to repeat the Pittsburgh Sleep Quality Index to reassess their own sleep. For cases, the severity of AD and symptoms of AD will be reassessed. Infant and parental quality of life will be reassessed. Treatment for AD will be amended, according to clinical response. Parental usage of products will be recorded.

Parents/guardians will be asked to complete monthly sleep diaries and questionnaires at 10 and 11 months to collect data on the infant’s 24-hour routine, focusing on naps and sleep routine, sleep environment (crib or parent’s room), sleep position (prone or supine), and need for sleep aids (e.g. soother, rocking, patting).

At 12 months, weight, length, and head circumference will be measured again. Parents will again be asked about specific sleep problems by thorough history of the infant’s 24-hour routine, focusing on naps and sleep routine, sleep environment (crib or parent’s room), sleep position (prone or supine), and need for sleep aids (e.g. soother, rocking, patting). Parents will be asked to repeat the Pittsburgh Sleep Quality Index to reassess their own sleep. Transepidermal water loss and natural moisturizing factor assessment will be repeated. Tape stripping for inflammatory cytokine analysis will be repeated. The severity of AD and symptoms of AD will be reassessed. Infant and parental quality of life will be reassessed. Treatment for AD will be amended, according to clinical response. Parental usage of products will be recorded. Each parent will be provided with a pair of sleep actigraphy pants with an embedded sensor belt to monitor overnight sleep movements every night for five nights following the visit.

At 18 months, subjects will be brought back to the INFANT Research Centre for their final study visit. At this visit weight, height, and head circumference will be measured. Mothers will be asked to fill out the Beck Depression Inventory. For cases, AD status and severity will be reassessed. In addition, a neurodevelopmental screening protocol will be completed to screen for behavioural difficulties and to ensure that all the participants are normally developing. This assessment will be performed using the CogniTOT touchscreen app (Liltoda, Cork, Ireland). CogniTOT is a touchscreen-based 10- to 15-minute cognitive assessment designed to screen for cognitive delay in young children aged 18-30 months with good concurrent validity with the Bayley’s Scales of Infant Development [53]. It will be suitable to use even if current social restrictions continue. Behaviour will be assessed using parental questionnaires. The Achenbach Child Behaviour Checklist 1 ½ - 5 is a widely-used questionnaire to assess behavioural and emotional difficulties, filled out by parents. It assesses internalizing (i.e., anxious, depressive, and over-controlled) and externalizing (i.e., aggressive, hyperactive, noncompliant, and under-controlled) behaviours. Several sub-areas are also measured, including anxiety and depression, and ‘Sleep Problems syndrome’. The Modified Checklist for Autism in Toddlers, Revised is a screening questionnaire for autism spectrum disorder. Any worrying or incidental findings will be referred for follow up along the relevant clinical pathway.

The primary outcome will be altered macrostructure of sleep at 12 months (sleep diary and actigraphy), measured as decreased sleep efficiency and increased night time awakenings. The secondary outcomes will be altered microstructure of sleep at six-eight months, measured as sleep spindle power and power in REM and NREM sleep stages; and parental sleep quality and quality of life. Outcomes will be correlated with severity of AD and inflammatory cytokines at six-eight and 12 months. In terms of safety outcomes, the clinical, neurophysiological and cytokine analyses are all safe and well-tolerated procedures with no significant associated risks. Potential confounding factors which will be controlled for in the data analysis will include parental educational level, parental depression, feeding practice, and number of siblings.

To calculate the sample size, an incidence of sleep disturbance of 60% in the infants with moderate to severe AD [17] and an incidence of 25% in healthy controls [54] will be assumed. Therefore, aiming for a power of 80% and a significance level of 5%, a total sample size of 30 subjects will be required for each group to power the dichotomous primary outcome of disturbed macrostructure of sleep and secondary outcome of disturbed microstructure of sleep. Assuming a worst-case attrition rate of 60%, and to ensure that data are available at all time points, 50 subjects will be required for each group, with a total number of participants of 100.

For descriptive statistics, mean ± standard deviation will be used, median and interquartile range for quantitative variables, and percentage with number for categories. Spearman (parametric) or Pearson (non-parametric) correlations will be applied to determine the association between dependent and independent (parental and infantile) variables. Independent variables will be fitted to logistic and linear regression models to evaluate interactions as well as to identify confounding factors. All the analyses will be carried out with the statistical software Stata, considering a *p* < 0.05 as cut-off for statistical significance. For sleep diaries, mean questionnaire scores and subsections will be compared between groups. For actigraphy, total number of overnight movements will be compared between groups. For EEG, the number, power, and density of sleep spindles, and sleep staging will be compared between groups. For parental sleep, mean questionnaire scores will be compared between groups, and qualitative parental interviews will be performed with a selection of parents of infants with AD. Qualitative data will be processed using NVIVO software.

Data will be recorded using the Castor Electronic Data Capture tool. Data will be stored on secure UCC servers. The screening and consent will take place within the first six-eight months of life at the recruiting site. Demographic details will be captured at six-eight months. Data on sleep quality will be obtained by EEG assessment during an afternoon nap at six-eight months and from overnight actigraphy recordings at six-eight months and 12 months. Data on sleep quantity will be obtained using parental sleep diaries and questionnaires from six to 12 months. Neurodevelopmental assessment will be performed at 18 months. Transepidermal water loss, natural moisturizing factor levels and skin cytokine analysis at six-eight months and 12 months will be recorded digitally. For cases, treatment logs will be kept in paper format to facilitate adjustments in treatment plans. Parents will be provided with a phone number and email address for the study to ensure easy access to the study team. Data will be entered directly onto the database by the study researcher. All data will be treated confidentially and held on a secure UCC server with restricted and password-protected access.

## Discussion

This study will focus on infants with early-onset AD who are at high risk of developing sleep disturbance. The study will investigate early qualitative and quantitative sleep changes using high quality sleep EEG, detailed parent-reported sleep data, and age-appropriate overnight actigraphy. EEG changes related to sleep have never been investigated in AD. The use of sleep pants for actigraphy also provides a novel method of assessing overnight movement in infants with AD, which is more age-appropriate than conventional actigraphy measures used in adults such as wrist sensors. The capture of monthly sleep data on infants with AD will provide details on the trajectory of sleep in the first year of life in AD. Data on the impact of AD on the sleep of both Mothers and Fathers will also be collected, which is important as infants with AD are wholly dependent on caregivers for management of their skin condition. The study will also correlate changes in the skin barrier and cutaneous immune profile to assess the relationship between them, using both novel and validated assessment tools. The findings will highlight the impact of AD on sleep and help to guide treatment plans for infants with early-onset AD.

The study has been designed to optimize retention and follow up by limiting the number of visits and by streamlining the follow-up questionnaires into a simple email survey link or application. Parents of children with AD are highly motivated to have their child’s AD treated well and will have easy access to expert dermatology care during their participation in the study. Parents of control infants will volunteer to participate. Previous studies in our research center and initial parent involvement in this study have suggested that parents are highly interested in involving their children in sleep research and that they consider the investigations easily tolerated. The final assessment at 18 months is a detailed neurodevelopmental assessment which is not currently available outside of the research setting and provides a rich assessment of multiple cognitive domains.

We will disseminate the results of this research via publication in high-impact peer-reviewed journals, and presentation at international conferences. The results will also be published in plain language summaries for the public and will be shared on social media.

This study will provide a detailed analysis of sleep in infants with AD in the first year of life. It may provide guidance on the optimal treatment of AD to prevent or reduce sleep disruption. It may also reveal causative pathways linking AD and sleep disruption to permit novel therapeutic strategies.

## Data Availability

The dataset generated from this study will be available on reasonable request from the corresponding author (COC). The data will not be publicly available due to consideration for information that could compromise research participant privacy or consent.

## Abbreviations

AD: Atopic Dermatitis
CUH: Cork University Hospital
CUMH: Cork University Maternity Hospital
EASI: Eczema Area and Severity Index
EEG: Electroencephalography
IGA: Investigator Global Assessment
INFANT: The Irish Centre for Maternal and Child Health Research
NMF: Natural moisturizing factor
REM: Rapid eye movement
SCORAD: SCORing Atopic Dermatitis
TEWL: Transepidermal water loss
UCC: University College Cork
